# Agonist muscle adaptation accompanied by antagonist muscle atrophy in the hindlimb of mice following stretch-shortening contraction training

**DOI:** 10.1186/s12891-017-1397-4

**Published:** 2017-02-02

**Authors:** Erik P. Rader, Marshall A. Naimo, James Ensey, Brent A. Baker

**Affiliations:** 1 Centers for Disease Control and Prevention, National Institute for Occupational Safety and Health, MS L3014, 1095 Willowdale Rd, Morgantown, West Virginia 26505 USA; 2 West Virginia University School of Medicine, Division of Exercise Physiology, Morgantown, West Virginia USA

**Keywords:** Stretch-shortening contractions, Plantarflexor muscles, Dorsiflexor muscles, C57BL6 mice, Myosin heavy chain, Muscle physiology, Biomechanics, Muscle fiber type

## Abstract

**Background:**

The vast majority of dynamometer-based animal models for investigation of the response to chronic muscle contraction exposure has been limited to analysis of isometric, lengthening, or shortening contractions in isolation. An exception to this has been the utilization of a rat model to study stretch-shortening contractions (SSCs), a sequence of consecutive isometric, lengthening, and shortening contractions common during daily activity and resistance-type exercise. However, the availability of diverse genetic strains of rats is limited. Therefore, the purpose of the present study was to develop a dynamometer-based SSC training protocol to induce increased muscle mass and performance in plantarflexor muscles of mice.

**Methods:**

Young (3 months old) C57BL/6 mice were subjected to 1 month of plantarflexion SSC training. Hindlimb muscles were analyzed for muscle mass, quantitative morphology, myogenesis/myopathy relevant gene expression, and fiber type distribution.

**Results:**

The main aim of the research was achieved when training induced a 2-fold increase in plantarflexion peak torque output and a 19% increase in muscle mass for the agonist plantaris (PLT) muscle. In establishing this model, several outcomes emerged which raised the value of the model past that of being a mere recapitulation of the rat model. An increase in the number of muscle fibers per transverse muscle section accounted for the PLT muscle mass gain while the antagonist tibialis anterior (TA) muscle atrophied by 30% with preferential atrophy of type IIb and IIx fibers. These alterations were accompanied by distinct gene expression profiles.

**Conclusions:**

The findings confirm the development of a stretch-shortening contraction training model for the PLT muscle of mice and demonstrate that increased cross-sectional fiber number can occur following high-intensity SSC training. Furthermore, the TA muscle atrophy provides direct evidence for the concept of muscle imbalance in phasic non-weight bearing muscles, a concept largely characterized based on clinical observation of patients. The susceptibility to this imbalance is demonstrated to be selective for the type IIb and IIx muscle fiber types. Overall, the study highlights the importance of considering muscle fiber number modulation and the effect of training on surrounding muscles in exercise comprised of SSCs.

**Electronic supplementary material:**

The online version of this article (doi:10.1186/s12891-017-1397-4) contains supplementary material, which is available to authorized users.

## Background

Exercise has been recognized as among the most effective interventions including nutrition at promoting wellbeing and alleviating chronic disease [1–4]. As reported in a review of meta-analyses, exercise, in particular, is as effective as drug treatment for the prevention of heart disease and diabetes, the treatment of heart failure, and the rehabilitation from stroke [5]. Resistance exercise, in particular, is being considered and/or encouraged to address muscle atrophy associated with such conditions such as cachexia, aging, and type 2 diabetes [1, 2]. Specifically, resistance exercise promotes adaptation in terms of greater muscular strength/power and muscle hypertrophy [2]. Such hypertrophy can come in the form of increased muscle fiber size and, in some cases, increased muscle fiber number [2, 6–11].

Daily activity and exercise (including resistance exercise) typically consist of movements requiring a sequence of different types of contractions, isometric, lengthening, and shortening contractions, i.e. SSCs [12]. To investigate the response to contractions with high control and precision, dynamometer based animal studies have been utilized [13]. The majority of these studies have investigated isometric, lengthening, or shortening contractions in isolation in order to characterize the impact of each contraction mode alone [13, 14]. An exception to this has been the study of SSCs in their entirety in a rat model [13, 15–17]. However, this animal model has been limited in regard to diversity of genetically defined strains available.

Mice are the most commonly used animal model for biomedical research. This is largely because they have biological similarities to humans, have been characterized as experimental models for nearly a century, and are available in thousands of genetically defined strains to aid in the investigation of molecular mechanisms (e.g. more than 7,000 genetically defined strains are maintained at Jackson Laboratories) [18]. Given these advantages, establishment of a SSC training protocol for mice would provide a valuable research tool for determining the molecular mechanisms of muscle adaptation.

The aim of the present study was to develop a dynamometer-based SSC training protocol to induce adaptation of muscle mass and performance gains for plantarflexor muscles of mice. We hypothesized that the plantarflexor muscles of mice would adapt to one month of training with sessions of 80 SSCs, a protocol based on that which has been demonstrated previously to be effective for hindlimb muscles of rats [15, 16, 19]. Consistent with the hypothesis, the 80 SSC protocol induced an increase in plantarflexion performance and PLT muscle mass. Additionally, unanticipated outcomes emerged which raised the value of the training model further. Following SSC training, the performance increase and agonist muscle mass gain was accompanied by an increase in muscle fiber number in transverse muscle sections while the antagonist muscle, the TA muscle, atrophied. These outcomes were not observed in previous research regarding the rat model [13, 15–17]. Furthermore, we characterized fiber type transitions and utilized the Myogenesis & Myopathy RT^2^ Profiler™ PCR Array to establish gene expression profiles for these plantarflexion SSC training induced alterations. The outcomes of this research establish this SSC training mouse model as an exceptional research model and provides insights into muscle fiber type transitions, fiber number modulation, and muscle imbalance.

## Methods

### Animals

A total of 18 male C57BL/6 J mice (Jax# 000664) at 3 months of age were obtained and housed in an Association for Assessment and Accreditation of Laboratory Animal Care (AAALAC)-accredited animal quarters. All animal procedures were approved by the Animal Care and Use Committee at the National Institute for Occupational Safety and Health in Morgantown, WV.

### Plantarflexion SSC training

Plantarflexor muscles were exposed to SSC training based on a previously validated procedure which induced muscle mass and performance gains in young rats [15, 16, 19]. For each SSC session, the mouse was anesthetized with isoflurane gas and placed in dorsal recumbency on a heated table. The particular anesthesia was selected because isoflurane does not have an observable effect on in vivo muscle force output [20]. The left foot was secured to a footplate of a dual mode muscle lever system (Whole Mouse Test System, 1300A, Aurora Scientific) allowing measurement of torque and control of foot rotation about the ankle. Platinum electrodes were placed subcutaneously to activate the tibial nerve for stimulation of the plantarflexor muscles. For all of the contractions, muscle stimulation was set at parameters for maximal contraction; 8-V magnitude, 0.2-ms pulse duration, and 150-Hz frequency.

Both static and dynamic performance was assessed prior to exposure to the 80 SSC session. Static performance was assessed by exposing the muscles to a single maximal isometric tetanic contraction for 200 ms with the ankle at 90° (angle between tibia and foot). After a 2-min rest, dynamic performance was assessed by exposure to a single SSC test consisting of an isometric contraction for 200 ms at an ankle angle of 110°, then rotating the ankle to 70° at 500° per second, then returning to 110° at 500° per second, and lastly continuing activation for an additional 200 ms. Peak force was determined by assessing the maximum force during the stretch phase.

The 80 SSC session was administered 2 min following the single SSC test. The protocol consisted of 8 sets of SSCs (2-min intervals between sets) and 10 SSCs per set (3-s intervals between SSCs) with the interval durations chosen so as to be comparable to those typically suggested for resistance exercise training [21]. For each SSC, while the muscles were maximally activated, the ankle was set to 90° for 100 ms, rotated to 70° at 60°/s, returned to 90° at 60°/s, and deactivated 100 ms later. Such a velocity of 60°/s promotes hypertrophy without overt muscle inflammation and degeneration in young rats in the days to weeks following chronic SSC exposure [15, 19, 22]. Following the 80 SSCs, a single maximal isometric tetanic contraction was measured at 5 min and a single SSC test was assessed at 7 min using the same parameters as those for these measures prior to the 80 SSC exposure. Comparison of these post- with pre-80 SSC exposure values provided a means to determine extent of recovery from fatigue [16]. Training consisted of 4 weeks of 2 to 3 days per week of SSC sessions. The performance measures for sessions during the first and last week of the SSC exposures were averaged to determine initial non-trained and final trained values, respectively. At 72 h after the final SSC exposure, hindlimb muscles were removed, weighed, and the tibia length recorded. For trained muscles and contralateral non-trained muscles, muscle mass was divided by tibia length to determine normalized muscle mass. The mid-belly of the muscles were covered with tissue freezing media (Tissue-Tek, Sakura Finetek) and frozen in cold isopentane (−80 °C) for quantitative morphology and immunoflurescence. A portion of the remaining tissue was allocated for gene expression analysis.

### Quantitative morphology

The mid-belly of each muscle was cryosectioned at 12 μm thickness. The cryosections were hematoxylin and eosin stained by the following protocol – 100% ethanol for 30 s, water for 1 min, Harris Hematoxylin for 3 min, water for 30 s, 0.1% acid alcohol for 30 s, water for 90 s, 80% ethanol for 30 s, Eosin Y for 1 min, 100% ethanol for 150 s, and Xylene for several dips. The sections were then analyzed by quantitative morphology using a standardized stereological method [17, 23, 24]. The investigator was blinded to sample identification during this analysis. The stereological method entailed identifying two non-overlapping regions to the right and left of the section midline. At each of these regions, stereological analysis was performed at 5 equally spaced sites across the muscle section. At each site, points of a 121-point 11-line overlay graticule (0.04 mm^2^ square with 100 divisions) were evaluated at 40× magnification [17]. Each point was identified as overlaying a muscle fiber (degenerative or non-degenerative) or interstitium (cellular or non-cellular). Degenerative muscle fibers were considered to have all of the following attributes: loss of contact with surrounding fibers, interdigitation of the sarcolemma by cellular infiltrates, and internalization of cellular infiltrates as apparent in the hematoxylin/eosin stained muscle sections [23]. If a muscle fiber lacked these characteristics, the fiber was considered to be non-degenerative. Cellular interstitium were counted when points overlaid nuclei in between muscle fibers. Points that overlaid interstitial regions without nuclei were counted as non-cellular interstitium. Percent of muscle tissue comprised of non-degenerative muscle fibers, degenerative muscle fibers, centrally nucleated muscle fibers, cellular interstitium, or non-cellular interstium were calculated as the percentage of points which overlaid each type of tissue relative to the total number of points. The number of muscle fibers were also counted when the topmost point of the fiber perimeter was within the boundary of the graticule and this was divided by the total area sampled to determine number of muscle fibers per unit cross-sectional area. Mean muscle fiber area (μm^2^) was determined by dividing the percent of tissue comprised of muscle fibers by fiber number per unit area.

### Gene expression analysis

Muscle tissue which was flash frozen and stored at −80 °C was retrieved and a ~20 mg portion was homogenized with a Minin-BeadBeater 8 (Biospec) while in a vial containing 1 ml of TRIzol and 1.0 mm zirconia beads (BioSpec, Cat#11079110zx). RNA was isolated using the RNAqueous Phenol-free total RNA Isolation Kit (Ambion, Cat# AM1912). cDNA was synthesized using the RT^2^ First Strand Kit (Qiagen, Cat# 330401). Samples were then analyzed using the Mouse Skeletal Muscle: Myogenesis & Myopathy RT^2^ Profiler™ PCR Array (Qiagen, Cat# PAMM-099Z) and RT^2^ SYBR Green Mastermix (Qiagen, Cat# 330523) per manufacturer’s instructions with use of an Applied Biosystems 7500 Real-Time PCR instrument. Fold changes and *P* values were determined from C_t_ values analyzed using the analysis template provided by Qiagen (PCRArrayDataanalysis_V4). Criteria for gene expression exceeding 1.3-fold change and *P* < 0.05 were used to identify differentially expressed genes. Bioinformatic analysis was performed for differentially expressed genes using Ingenuity Pathway Analysis (IPA; Ingenuity Systems, www.ingenuity.com). IPA biological functions with the exception of cancer specific functions were evaluated.

### Fiber type analysis

The transversely cryosectioned mid-belly of each muscle underwent immunofluorescence myosin heavy chain (MHC) staining using a previously described method [16]. Sections were blocked with 10% goat serum in PBS for one hour at room temperature and then incubated in the following primary antibody cocktail overnight at 4 °C – antibodies against MHC I (BA-F8; 1:10), MHC IIa (SC-71; 1:200), MHC IIb (BF-F3;1:200), and laminin (L9393;1:400). MHC antibodies were purchased from Developmental Studies Hybridoma Bank (University of Iowa) and the laminin antibody was purchased from Sigma. A cocktail of secondary Alexa Fluor antibodies (Life Technologies) were applied for two hours at room temperature – 350 IgG2b goat anti mouse (A21140; 1:250), 594 IgG1 goat anti-mouse (A21125; 1:100), 488 IgM goat anti-mouse (A21042; 1:500), and 488 IgG goat anti-rabbit (A11008; 1:500).

Fiber type analysis was performed by a standardized stereological method [15, 16]. With the investigator blinded to sample identification, stereological analysis was systematically repeated at 5 equally spaced sites across the section on each side of the midpoint. At each site, points of a 121-point 11-line overlay graticule (0.04 mm^2^ square with 100 divisions) were evaluated at 20× magnification. Since 121 points were evaluated in 10 fields, a total of 1210 points were analyzed per muscle section. Each point was identified as overlaying a MHC I (blue), MHC IIa (red), MHC IIb (green), MHC IIx (lacking staining), and interstitium. The number of muscle fibers corresponding to each fiber type were also counted when the topmost point of the fiber perimeter was within the boundary of the graticule. The total number of fibers counted divided by the total area sampled over the 10 regions (i.e. 0.4 mm^2^) was calculated to determine the number of fibers per unit cross-sectional area (number of fibers per mm^2^). The percentage of each fiber type was determined by dividing the number of fibers corresponding with each fiber type by the total number of fibers counted. Mean muscle fiber area (μm^2^) for a particular fiber type was determined by dividing the percent of tissue comprised of that fiber type by the appropriate fiber number per unit area.

### Statistical analysis

Data were analyzed using paired Student’s t-tests or ANOVA (JMP version 11, SAS Institute, Inc., Cary, NC) with the variable of animal identification as a random factor to account for repeated measures when appropriate. *Post hoc* comparisons were performed using Fisher’s least significant difference method. When normality could not be assumed, data were analyzed with the Wilcoxon Signed Rank Test. Chi-square analysis (SigmaPlot version 12.5, Systat Software, Inc., San Jose, CA) was utilized to determine training-induced differences in the absolute frequency distributions of fiber type. All data are expressed as means ± SE. *P* < 0.05 was considered statistically significant.

## Results

### Effective SSC training for enhancement in plantarflexion performance accompanied by differential responses in muscle mass PLT and TA muscles

**Fig. 1:**
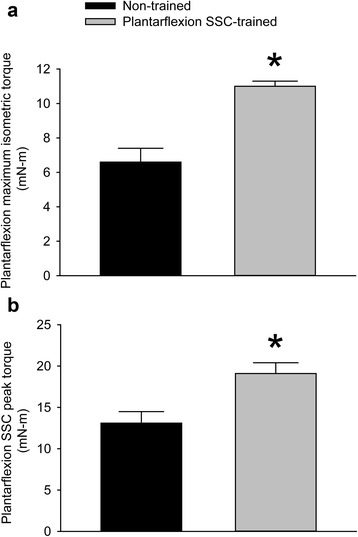
Plantarflexion SSC training enhanced plantarflexor muscle performance. Values of **a** static performance in terms of maximum isometric torque and **b** dynamic performance in terms of peak torque during a single SSC test were assessed. Sample sizes were *N* = 9 per group. Values are means ± SE. *Different from non-trained value, *P* < 0.05

**Fig. 2:**
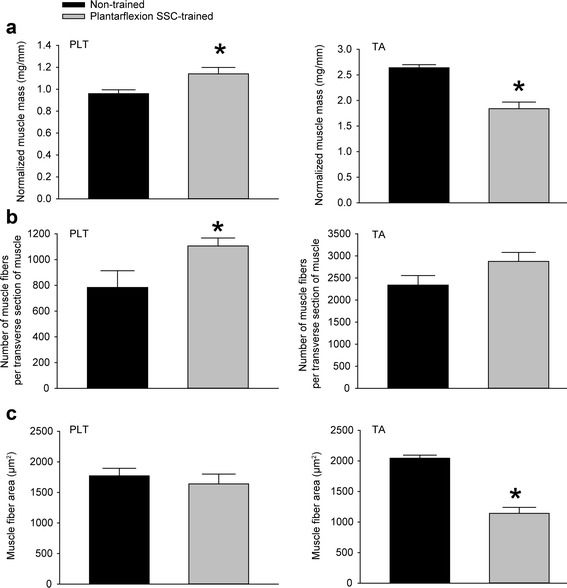
The agonist PLT muscle increased muscle mass with plantarflexion SSC training whereas the antagonist TA muscle atrophied. Values for **a** muscle mass (normalized by tibia length) **b** number of muscle fibers per transverse muscle section, and **c** mean muscle fiber area were determined. For PLT muscles, an increase in the number of muscle fibers in cross-section accompanied the increase in mass. For TA muscles, atrophy was observed at the individual muscle fiber level. *N* = 9 per group with exception of total muscle fiber counts for PLT muscles. A subpopulation of all the PLT muscle sections (*N* = 3 to 8 per group) fulfilled the requirement for direct fiber counts – the requirement that all regions of the muscle section be free of technical defects typically incurred from prepping and cryosectioning such a relatively small muscle. Other measures (e.g. muscle fiber area) were obtained from all of the PLT muscle sections (*N* = 9 per group) because these relied on the sampling approach of stereological quantitative morphology. Values are means ± SE. *Different from non-trained value, *P* < 0.05

Plantarflexion SSC training increased static performance two-fold in terms of maximum isometric torque and dynamic performance in terms of peak torque during the SSC test (Fig. 1a and b). This increase in plantarflexion performance was accompanied by a 19% increase in PLT muscle mass and a 30% decrease in TA muscle mass with no change in mouse body weight, 25.6 ± 0.6 g in the initial non-trained state and 25.3 ± 0.6 g after training (Fig. 2a). Training had no effect on gastrocnemius and soleus muscle mass. Normalized muscle masses for trained gastrocnemius and soleus muscles, 7.16 ± 0.18 and 0.48 ± 0.04 mg/mm, respectively, were not different from values for contralateral muscles, 7.38 ± 0.15 and 0.44 ± 0.05 mg/mm. Investigation at the muscle fiber level revealed distinct training induced alterations accounting for the changes in muscle mass for the PLT and TA muscles. The number of muscle fibers per transverse muscle section increased for PLT muscles while no effect of training was observed for TA muscles (Fig. 2b). The mean muscle fiber area decreased for TA muscles with no such effect for PLT muscles (Fig. 2c). Therefore, the results indicated that the increased muscle mass for PLT muscles was due to the addition of muscle fibers in cross-section while the decreased muscle mass for TA muscles was due to muscle fiber atrophy.

**Fig. 3:**
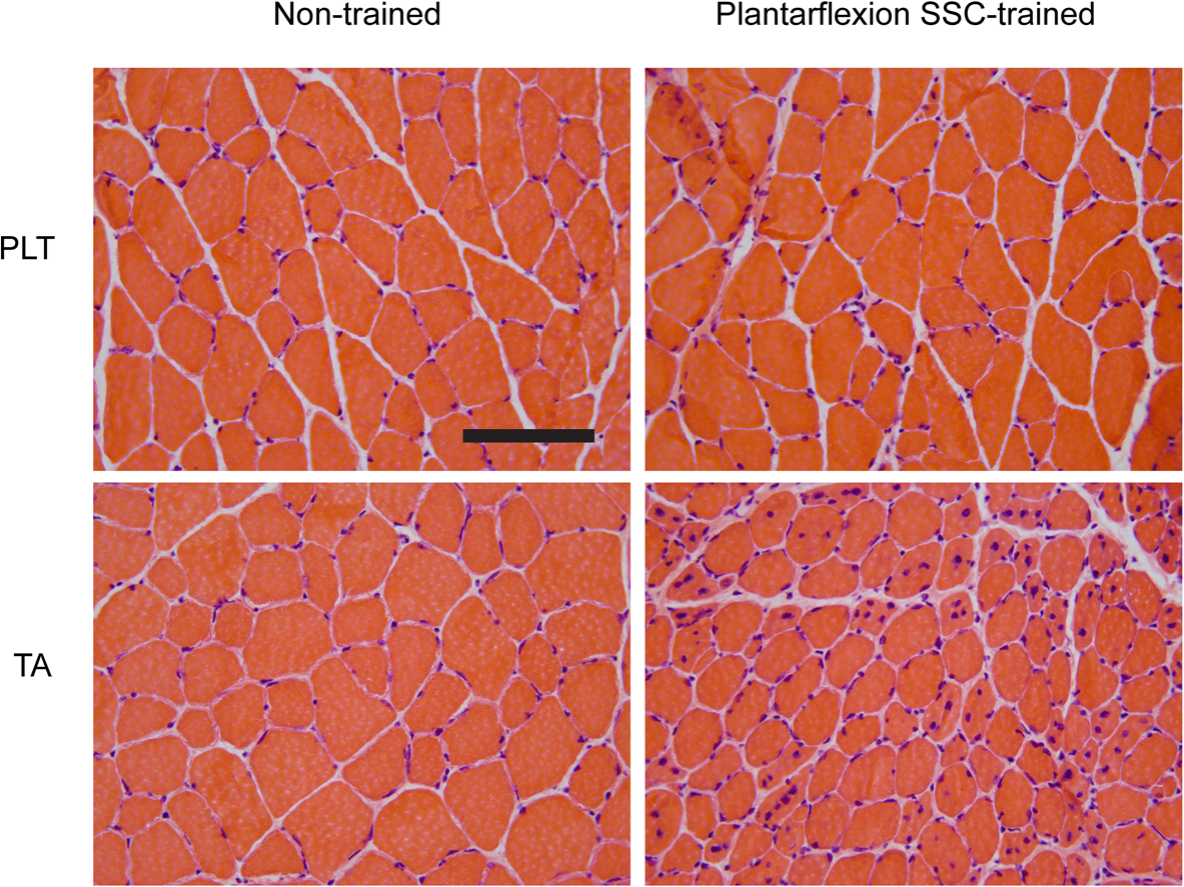
Altered muscle fiber morphology was most noticeable in TA muscle sections following plantarflexion SSC training especially in terms of an increased proportion of centrally nucleated muscle fibers. Transverse sections of PLT and TA muscles were stained with hematoxylin and eosin. Scale bar = 100 μm

Hematoxylin and eosin stained muscle sections were then evaluated for extent of overt degeneration in muscle fibers and presence of cellular constituents in the interstitium (Fig. 3). For both muscles, no significant extent of muscle fiber degeneration was observed, a finding consistent with the absence of an overt inflammatory response (Table 1). The muscles differed in the extent of training-induced alterations in the interstitium and prevalence of centrally nucleated fibers. For PLT muscles, the effects of training were limited to the interstitium in that the percentage of tissue composed of non-cellular interstitium and cellular interstitium increased (Table 1). However, these increases were marginal considering that the percentage of tissue consisting of muscle fibers was unaltered. This was not the case for TA muscles. For these muscles, the training-induced increases in the composition of non-cellular interstitium and cellular interstitium were sufficiently increased enough to account for a decrease in percentage of tissue composed of muscle fibers. In addition, an increase in percentage of centrally nucleated fibers was observed (Table 1 and Fig. 3).

**Table 1:** Quantitative morphology for muscles of mice exposed to plantarflexor SSC training

	Non-trained	Plantarflexion SSC-trained
PLT		
Non-degenerative muscle fibers (%)	86.7 ± 0.9	84.5 ± 0.5
Degenerative muscle fibers (%)	0.0 ± 0.0	0.0 ± 0.0
Non-cellular interstitium (%)	12.2 ± 0.7	14.3 ± 0.5*
Cellular interstitium (%)	0.8 ± 0.1	1.3 ± 0.1*
Centrally nucleated fibers (%)	4.0 ± 0.9	14.0 ± 5.2
TA		
Non-degenerative muscle fibers (%)	88.2 ± 0.5	83.2 ± 1.4*
Degenerative muscle fibers (%)	0.0 ± 0.0	0.0 ± 0.0
Non-cellular interstitium (%)	11.1 ± 0.5	15.2 ± 1.3*
Cellular interstitium (%)	0.7 ± 0.1	1.6 ± 0.2*
Centrally nucleated fibers (%)	1.5 ± 0.7	17.5 ± 5.7*

Sample sizes were *N* = 9 per group. Percentage refers to percentage of tissue. Values are means ± SE. *Different from non-trained muscle, *P* < 0.05

### Gene expression analysis indicating muscle specific regulation of myogenesis, atrophy/hypertrophy, energy metabolism, and skeletal muscle fiber type

To investigate the differential responses in PLT and TA muscles further, gene expression of 84 genes was analyzed using the Mouse Skeletal Muscle: Myogenesis & Myopathy RT^2^ Profiler™ PCR Array. For PLT muscles, 24 genes were differentially expressed, grouped based on gene function according to the PCR array manufacturer’s recommendations, and analyzed by IPA to determine highly predicted biological functions (Additional file 1: Tables S1, Additional file 2: Table S2, Additional file 3: Table S3, Additional file 4: Table S4 and Additional file 5 Table S5). Hypertrophy was the most predicted function to be upregulated. The prediction in IPA was largely based on the transcriptional response in autocrine/hypertrophy signaling genes (e.g. *Mstn*), and the downregulation of genes which can promote atrophy (e.g. *Fbxo32*, *Foxo1*, and *Hdac5*). Consistent with muscle hypertrophy as a consequence of increased fiber number in cross-section, the fusion of cells and quantity of muscle cells were among the highest predicted biological functions. IPA attributed enhanced fusion of cells to the upregulation of *Tgfb1*, *Myf5*, *Myog*, and *Igf2* and downregulation of *Foxo1* while the prediction of increased muscle cell quantity was attributed to the upregulation of *Myog* and *Pax7* and downregulation of *Foxo1* and *Mstn*. In regards to energy metabolism, IPA predicted a training induced shift away from glycolysis based on the downregulation of *Hk2*, *Slc2a4*, *Pdk4* and upregulation of *Myog*. Consistent with this, was the indication of an alteration in fiber type distribution with the downregulation of *Tnni2*, *Tnnt3*, and upregulation of *Myh1*.

A total of 27 genes were differentially expressed for TA muscles following plantarflexion SSC training (Additional file 1: Tables S1, Additional file 2: Table S2, Additional file 3: Table S3 and Additional file 4: Table S4). Cell death was among the most highly predicted to be increased by IPA (Additional file 5: Table S5). In IPA, this prediction was attributed largely to upregulation of *Casp3* and *Lmna* and downregulation in *Rps6kb1*, *Pax3*, and genes for proteins relevant to dystroglycan and titin (*Dag1*, *Ttn*, *Capn3*, and *Camk2g*). The downregulation of genes for dystroglycan and titin complex-related proteins also resulted in the prediction of decreased skeletal muscle function.

Atrophy of muscle was another highly predicted function largely based on the upregulation of *Casp3* and downregulation in *Ppargc1a* and *Ttn*. Apoptosis appeared relevant considering the prediction for the category of apoptosis of neurons based on upregulation of *Casp3* and *Musk* and the downregulation of *Pargc1a* and *Fgf2*. Several predicted functions (e.g. glucose metabolism disorder, hyperglycemia, and transport of D-glucose) suggested the development of a metabolic disorder for TA muscles consistent with the downregulation of *Ppargc1a*, *Rps6kb1*, *Slc2a4*, and *Prkab2*. The downregulation of *Tnni2, Tnnt3, and Myh2* suggested training-induced fiber type transitions also occurred for TA muscles. Overall, the gene expression analysis indicated plantarflexion SSC training-induced shifts in fiber type distribution along with opposing outcomes in terms of hypertrophy/atrophy, muscle function, and metabolism for PLT and TA muscles.

### Improved energy metabolism for PLT muscles confirmed by enhanced fatigue recovery

**Fig. 4:**
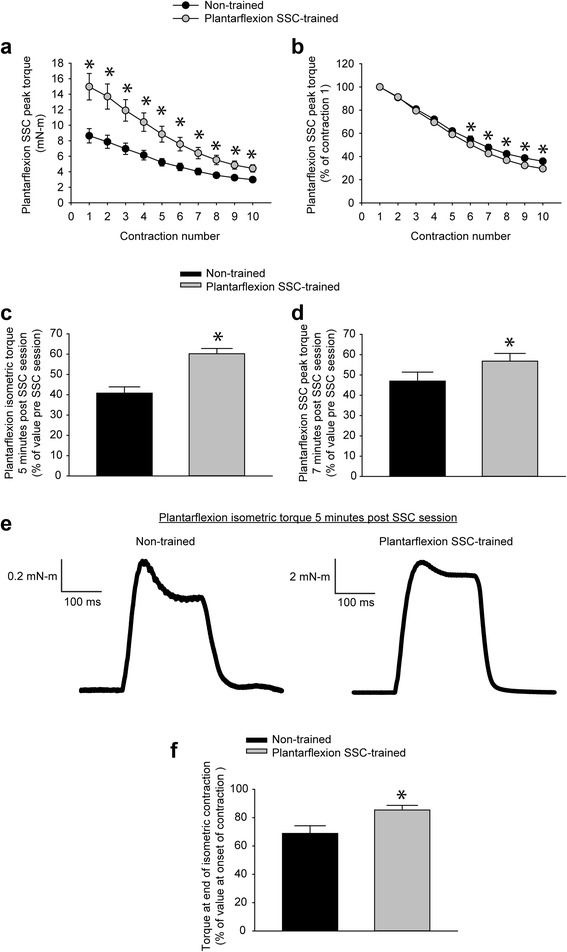
Plantarflexion SSC training improved plantarflexion recovery from fatigue. Values for peak torque were evaluated for the first set of 10 contractions. **a** With training, absolute values improved for all contractions of the first set. **b** When peak torques were normalized to that of the first contraction, training had no effect early in the set followed by a marginal effect late in the set. To assess recovery from fatigue, **c** an isometric contraction and **d** a single SSC test were assessed 5 minutes and 7 minutes after a training session of 80 SSCs, respectively. These values were expressed relative to their pre-session values. **e** For the isometric contraction following a session of 80 SSCs, maximum torque production typically could not be completely maintained for the duration of the contraction. However, a training-induced improvement in the degree to which torque could be maintained was noticed by reference to raw torque traces. **f** The extent to which torque was maintained during a post-session isometric contraction was quantified by expressing the torque at the termination of muscle activation as a percentage of the maximum torque value at the onset of contraction. Sample sizes were *N* = 9 per group. Values are means ± SE. *Different from non-trained value, *P* < 0.05

To determine whether the gene expression in PLT muscles regarding energy metabolism was consistent with muscle performance, functional data was evaluated further in terms of plantarflexion fatigue and recovery from fatigue. Fatigue was investigated during the first set of contractions in each SSC session. Training increased absolute peak torque values for all of the contractions of the first set (Fig. 4a). When these torque values were normalized to the first contraction of the session, only a marginal training-induced difference was observed for the final five contractions of the set (Fig. 4b). Therefore, the plantarflexor muscles adapted so that the rate of fatigue remained largely unchanged despite an increased metabolic demand concomitant with greater torque production.

Recovery from fatigue was evaluated for both the isometric contraction and the single SSC test contraction several minutes following each SSC session. Training improved the extent to which both of these measures returned to pre-session values (Fig. 4c and d). A training-induced change was observed for individual post-session isometric torque traces (Fig. 4e). Traces for non-trained muscles exhibited a 31% decrease in torque during the contraction while trained muscles had only a 15% decline (Fig. 4 f). The implication was that training improved fatigue recovery as evident by the maximum torque values for post-session contractions and by an improved ability to maintain torque within these contractions.

### Differential response in fiber type distribution and size for PLT and TA muscles

**Fig. 5:**
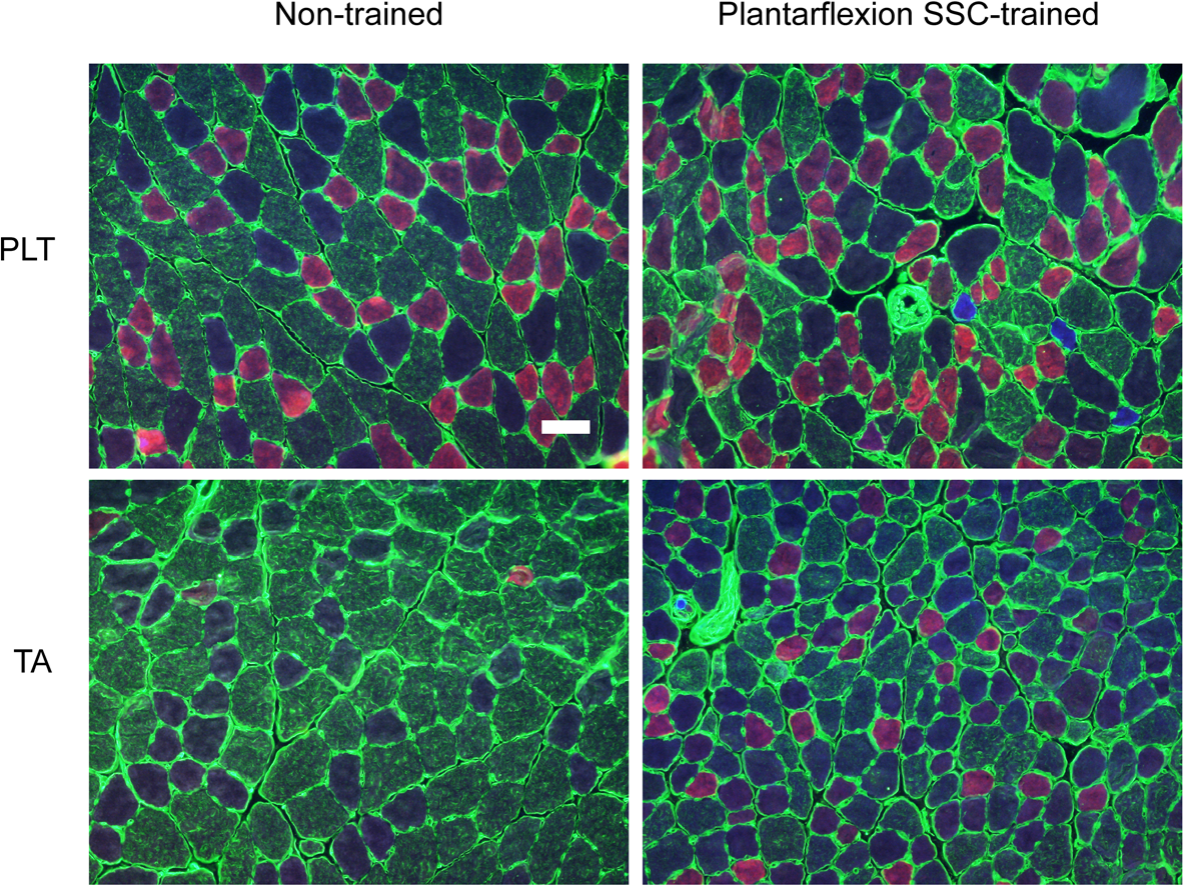
Differential fiber type distribution and size outcomes following plantarflexion SSC training for PLT and TA muscles. Laminin (*green*) and multiple MHC isoforms – I (*blue*), IIa (*red*), IIb (*green*) and IIx (negative for staining) – were identified by immunofluorescence. For PLT muscles, a shift from IIb to IIx fibers was apparent with no overt type II fiber size changes. For TA muscles, a shift from type IIb to IIx and IIa fibers accompanied by decreases in fiber sizes especially for type IIb and IIx fibers were noticeable. Scale bar = 50 μm

**Fig. 6:**
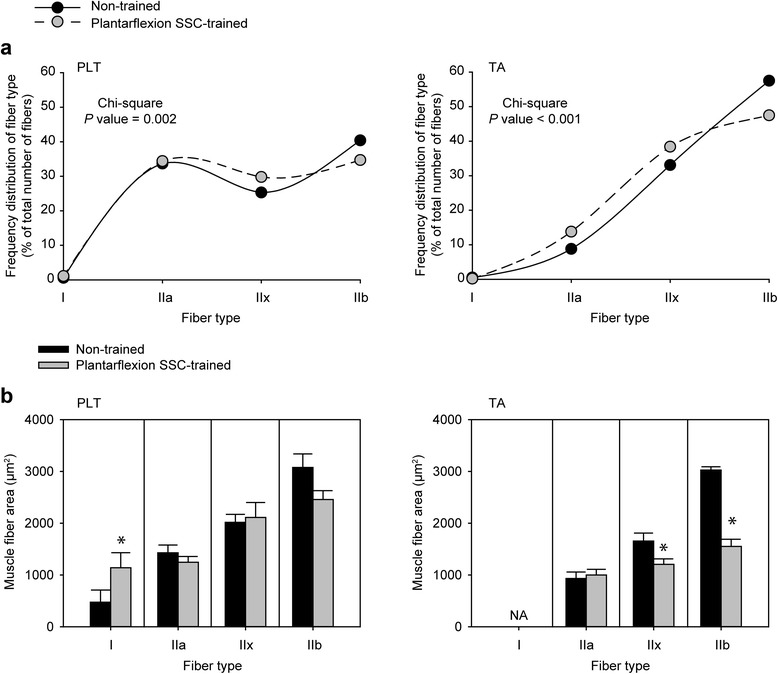
Quantification of fiber type distribution and size for PLT and TA muscles. **a** Frequency distributions for fiber type are presented as percentage of total number of fibers counted for each group (*N* = 9 muscles per group). Chi-square analysis was performed to determine alterations in distribution with training. **b** Mean muscle fiber size for each fiber type. Values are means ± SE. *Different from non-trained value, *P* < 0.05

Fiber type analysis was performed to investigate a potential mechanism for enhanced fatigue recovery in PLT muscles (Fig. 5). For PLT muscles, a shift from type IIb to type IIx muscle fibers was observed (Fig. 6a). Consistent with muscle fiber size data determined from analyzing hematoxylin and eosin stained sections (Fig. 2c), there were no training-induced changes in fiber area with the exception of an increase observed for type I fibers, fibers which make up only a marginal percentage of all fibers in PLT muscles (Fig. 6b). Therefore, overall, muscle fibers of PLT muscles shifted to a more oxidative phenotype without muscle fiber atrophy, a finding consistent with improved fatigue recovery capability in the context of muscle adaptation.

The muscle fiber type results for TA muscles were distinct from those for PLT muscles. Training induced a decrease in percentage of type IIb fibers accompanied by increases in the percentage of both type IIx and IIa fibers (Fig. 6a). This response was concomitant with decreases in muscle fiber size for both type IIb and IIx fibers. Such decreased size in the most prevalent muscle fiber types, IIb and IIx fibers, without an effect on type IIa fiber size demonstrated the selective nature of the atrophy in TA muscles induced by plantarflexion SSC training.

## Discussion

In the present study, we developed a novel adaptive SSC protocol for plantarflexor PLT muscles of mice, thereby effectively addressing the aim and hypothesis of the study in establishing in mice a practical and influential model for the study of SSC training. In developing this model, two major findings were observed. First, a shift in fiber type distribution accompanied by increased total fiber number (rather than enlarged fiber size) in cross-section as responsible for the increase in PLT muscle mass. This result highlights the present study as providing a unique model of SSC-induced adaptation given that our previous research regarding SSC training in rats demonstrated the more typical response of muscle fiber hypertrophy concomitant with increased muscle mass [19]. The second major finding of the study was the 30% decrease in the antagonist TA muscle mass with selective atrophy of type IIb and IIx fibers. Such an induced muscle imbalance indicates the profound detrimental effects of limiting resistance-type training to specific muscles without regard to surrounding muscles. Overall, the results have impact on understanding SSC-induced adaptation in regards to implementation in the mouse model, fiber type transitions, fiber number modulation, and the induction of muscle imbalance.

In developing this SSC training for the induction of increased plantarflexion torque and muscle mass, the serendipitous consequence of a shift to a more oxidative metabolism was evident by analysis of fatigue recovery, gene expression, and fiber type data. Increased maximum isometric force recovery has been observed previously for muscles of rats exposed to contraction-induced training [16, 25, 26]. In this study, we demonstrate that not only did fatigue recovery improve in terms of maximum isometric force but also capacity to maintain force generation during the entirety of a contraction shortly following a SSC session improved with training. Interestingly, resistance to fatigue during the first set of SSCs was largely unaltered with training. A comparable result of a shift away from type IIb fibers and differential adaptation for enhanced fatigue recovery rather than fatigue resistance has been observed for SSC-trained rats and endurance trained human subjects [16, 27]. This supports the concept that recovery from fatigue, rather than the susceptibility to fatigue, presents as more sensitive to certain training-induced alterations in fiber type [16, 27]. The training-induced alteration in metabolism for PLT muscles was apparent at the transcriptional level. A shift away from glycolysis was evident by the downregulation of genes for Slc2a4 (Glut4; glucose transporter), Hk2 (prepares glucose for glycolysis), and AMPK (a regulator of *Glut4* and *Hk2* expression) [28–30]. The upregulation of *Myog* may underlie this metabolic alteration. *Myog* overexpression in mice induces a shift from glycolytic to oxidative metabolism [31, 32]. At the fiber type level, the metabolic alteration of the present study is further supported with the shift from type IIb to IIx fibers and the upregulation of *Myh1,* coding for MHC IIx.

The observation of increased fiber number per PLT muscle cross-section was a novel finding in an animal model of SSC training. For the SSC training model in rats, increased muscle fiber size can account for the induced muscle hypertrophy [19]. With training, mean fiber size for rats of that study increased by 14% (2867 ± 107 vs. 2521 ± 84 μm, trained vs non-trained, *P* = 0.01) while fiber number did not (16,227 ± 529 vs. 15,966 ± 646 fibers, trained vs. non-trained, *P* = 0.65) [19]. This differential outcome may be due to differences in the mechanical strain of the SSC training protocols in terms of the extent muscles stretched and shortened each contraction. For the protocol utilized previously, muscles were stretched and shortened through an ankle rotation of 50° [15–17, 19]. Because of limitations in the muscle lever system of the present study, the ankle rotated through a decreased excursion (20° of ankle rotation). Consequently, muscles were exposed to SSCs which were more moderate when compared with those of previous studies. The finding that only PLT muscles increased in muscle mass while the other plantarflexor muscle masses were unaltered is consistent with the moderate nature of the training. Based on the glycolytic fiber type characteristics of the PLT muscle relative to the other plantarflexor muscles (soleus and gastrocnemius), the PLT muscle is recruited less often during daily activity and, consequently, activation during SSC training would be especially novel and sensitive to adaptation [33].

Evidence for contraction-induced increases in muscle fiber number has been observed in several studies regarding voluntary resistance training in animal models and human subjects [6–11]. The present study demonstrates that such a response can occur after high-intensity SSC training consisting of maximal activation of muscles. This increased fiber number potentially could have been mediated by the downregulation of *Mstn*. Postnatal muscle-specific inactivation of *Mstn* results in increased cross-sectional fiber number accounting for ~30% of the increase in whole muscle section area [34]. Furthermore, a missense mutation in *Mstn* causes increased muscle fiber number without fiber hypertrophy [35]. Consistent with the downregulation of *Mstn* in the present study, *Igf2* was upregulated. Myostatin is a negative regulator of *Igf2* expression [36]. Despite the common belief that *Igf2* has only a marginal role during postnatal muscle growth, recent studies have indicated otherwise [36, 37]. Pigs with a SNP within intron 3 of the paternal *Igf2* allele have increased postnatal *Igf2* expression and greater muscle mass [37, 38]. High postnatal levels of *Igf2* also accompany the increased muscle mass of *Mstn* null mice [36]. In addition to gene expression consistent with increased fiber number, the gene expression analysis was indicative of enhanced muscle fiber survival as evident by the downregulation of genes involved in protein degradation. Several genes associated with Fbxo32 (Atrogin-1) induced protein degradation (via the ubiquitin-proteasome pathway) were downregulated - *Fbxo32*, *Foxo1*, *Myod1*, and *Hdac5* [39, 40]. *Capn3,* the gene encoding the calpain 3 protease involved in an alternative proteolytic pathway, was also downregulated [40].

Whether the fiber number increase of the present study represents a completely different form of adaptation than that observed in the SSC trained rat model or characterizes an earlier stage of adaptation remains unknown. For instance, SSC training may induce first a fiber number increase, as observed in the present study, and then fusion of fibers (e.g. fusion of myofibers or branched fibers) with a return to control value fiber numbers, as observed in previous research regarding the rat SSC model [15–17, 19, 41]. This order of adaptation has been proposed as a possibility by others during regeneration following muscle injury [41, 42]. The present study is distinctive in suggesting that such a sequence of adaptation may also occur for muscles exposed to non-injurious training.

The concept of muscle imbalance has been characterized as hypertrophy or high recruitment of one muscle or muscle group as linked to an atrophy response in opposing muscles [43, 44]. Muscle imbalance has been implicated in underlying such conditions as lumbopelvic instability, lower back pain, susceptibility to knee injury, and shoulder pathology [43, 45, 46]. Based on observations of patients, phasic muscles (rather than tonic postural muscles) have been considered susceptible to possible atrophy and weakness from muscle imbalance [44]. Our findings regarding the phasic non-weight bearing TA muscles provide direct evidence for this concept. Furthermore, the present study establishes a gene expression profile and fiber type phenotype for muscle imbalance. The severity of the TA muscle atrophy was apparent at the gene expression level as evident by the downregulation for genes encoding proteins associated with titin and dystroglycan, proteins essential for force transmission during contractions [47]. Such downregulation suggested that the muscle was compromised in terms of quality (decreased expression of genes for cytoskeletal/sarcomeric proteins per unit of muscle mass) as well as quantity (i.e. overall muscle mass decrement). The depreciated state of the TA muscle was also evident by the upregulation of *Cryab*, an opposite change of regulation to that of the PLT muscle. *Cryab* encodes αB chain crystallin (i.e. heat shock protein B5), a molecular chaperone that binds misfolded proteins to prevent the accumulation of protein aggregates and is upregulated in stress/pathological conditions [48]. Gene expression analysis indicates that such a compromised condition in TA muscles was accompanied by the upregulation of gene expression promoting muscle atrophy (e.g. *Casp3*) and downregulation of genes which repress such atrophy (e.g. *Pax3* and *Rps6kb1*) [49, 50]. The downregulation of *Rps6kb1* was especially noteworthy given that *Rps6kb1* encodes p70 Ribosomal protein S6 kinase 1, a protein intimately linked to protein synthesis via regulation of major components of translation - Ribosomal S6 protein, eukaryotic translation initiation factor 4B, and eukaryotic elongation factor 2 [50, 51]. This indicated that the gene expression profile was consistent with decreased protein synthesis in addition to enhanced atrophy.

A neuromuscular imbalance may underlie the atrophy response for the TA muscle [44]. One instance of an onset of such an imbalance is when antagonist muscle activity is reduced secondary to training-induced tension of the agonist [52, 53]. This response contributes to the increased force production in trained agonist muscles. However, if antagonistic muscle inhibition is pervasive enough to be prevalent (even during daily activity between training sessions) such a response could be detrimental to antagonistic muscles. The notion of decreased antagonist activity is supported by the gene expression of *Myog* and *Musk*. Muscle-specific receptor tyrosine kinase (Musk) signals clustering of acetylcholine receptors and muscle activity suppresses expression outside of the neuromuscular junction [54]. When activity is abolished by denervation, myogenin upregulates *Musk* in an embryonic pre-innervation pattern of gene expression [54]. In the present study, *Myog* and *Musk* were highly upregulated (9- and 2-fold increases, respectively), a finding consistent with diminished TA muscle activity. Preliminary research regarding dorsiflexion SSC training also supports the notion that decreased muscle activity may underlie the antagonistic atrophy of the present study.^.^ Following dorsiflexion SSC training of young (3 month old) Sprague Dawley rats (*N* = 4), TA muscles, which are agonists in this case, increase normalized muscle mass by the expected 20% (trained vs non-trained; 17.3 ± 0.6 vs 14.4 ± 0.7 mg/mm; *P* = 0.03). Interestingly, for the same animals, such training had no effect on antagonist muscle masses in this situation, the tonic weight-bearing plantarflexor muscles (trained vs non-trained; PLT – 8.3 ± 0.2 vs 8.3 ± 0.2 mg/mm; gastrocnemius - 39.3 ± 1.2 vs 39.6 ± 1.3 mg/mm; and soleus - 3.3 ± 0.2 vs 3.4 ± 0.1). When these results are considered along with the present study, phasic non-weight bearing phasic TA muscle may be largely inhibited without significant impact on daily activity as in the present study while plantarflexor muscles may be protected from severe inhibition because of their weight-bearing activity in the preliminary study.

The shift away from MHC IIb observed for TA muscles in the present study is consistent with several reports regarding the MHC distribution alteration observed for fast-twitch muscles (e.g. TA and laryngeal muscles) following immobilization, nerve transection, and toxin-induced muscle paralysis [55–58]. Although the distribution alteration away from MHC IIb is in common with that of the PLT muscles, the downregulation of *Ppargc1a* suggests a diminished metabolic capacity for TA muscles after plantarflexion SSC training. *Ppargc1a* encodes peroxisome proliferator-activated receptor gamma coactivator-1α (PGC-1α), a master regulator of mitochondrial biogenesis and oxidative enzymes [59]. The downregulation of *Ppargc1a* coupled with a shift from MHC IIb fibers to MHC IIx and MHC IIa fibers suggests metabolic changes without the typical MHC alteration. A disconnect between MHC composition and metabolic capacity has already been shown to be possible previously for transgenic mice which overexpress myogenin [31]. The transition away from type IIb fibers in the present study was accompanied by atrophy of IIb and IIx fibers while type IIa fiber size was preserved. This tendency for a greater propensity for type IIb/x to atrophy has also been observed following immobilization of TA muscles, a finding consistent with specific catabolic conditions [60, 61]. Therefore, the atrophy of TA muscles in the present study was a function of both a shift in fiber type distribution (from large IIb fibers to smaller IIx and IIa fibers) and atrophy within fiber type, specifically for type IIb and IIx fibers.

The plantarflexion SSC training induced 30% atrophy of the TA muscle is striking given that this occurred at a young age, an age with high adaptive capacity and resistance to maladaptation as evident in resistance training for human subjects [62, 63]. Likewise in a previous report, when muscles of young rats were directly exposed to a severe SSC protocol – a protocol which induced performance deficits at maturity and old age – the response was muscle hypertrophy and enhanced performance [16]. However, these past studies were limited in that only agonist muscles were evaluated. The present study demonstrates that antagonist muscles are more susceptible to such maladaptation following a distinct SSC protocol; and, therefore, supports the concept of investigating antagonist and surrounding muscles in addition to the agonist muscle when evaluating training regimes. Such investigation is especially warranted because muscle imbalance can be largely subclinical until severe joint mobility, maladaptation, or possibly injury manifests. General activity/training alone is not sufficient to prevent this maladaptation as evident by studies demonstrating that elite athletes incur muscle imbalance [43, 46].

Overall, the outcomes of the present investigation effectively address the rational and aim of the study – to develop a dynamometer-based SSC training protocol to induce muscle mass and performance gains for the plantarflexor PLT muscle of mice. Regarding the major finding of increased muscle fiber number per PLT cross-section, a limitation was not being able to determine the precise mechanism (i.e. muscle fiber splitting vs *de novo* fiber formation) with the available data. Despite this, the finding establishes that increased fiber number is a possible response to high-intensity SSC training and warrants further investigation. The other major finding of the study - plantarflexion SSC training induced muscle imbalance in the antagonist TA muscle of mice - provides the scientific community with a novel model to investigate muscle imbalance. In addition, the finding is consistent with the proposal that antagonist phasic muscles are susceptible to imbalance when agonist muscles are exposed to contractions in isolation [43, 44]. Fortunately, such training-induced muscle imbalance is not inevitable. Research regarding volitional weight-lifting for rats demonstrates that squat-like training induces hypertrophy in the agonist plantarflexor muscles without atrophy of the antagonist TA muscle, a muscle activated for stabilization during squats [64]. Resistance-type training requiring antagonist stabilization should be considered as a potential strategy to prevent the occurrence of muscle imbalance. The further development of concepts for exercise prescription are justified to ensure the prevention of such deleterious outcomes as those observed for the phasic antagonist muscle of the present study.

## Conclusions

In summary, the present study establishes a dynamometer-based SSC training protocol to induce gains in performance and PLT muscle mass for mice, an underrepresented animal model for the study of SSC-induced adaptation. The major finding of a SSC-induced increase in cross-sectional muscle fiber number rather than muscle fiber size provides an impetus for the scientific community to rethink the pervasiveness of muscle fiber hypertrophy vs fiber number modulation following resistance-type training. A greater understanding of fiber number modulation has potential clinical relevance in countering conditions associated with muscle fiber loss such as aging, muscular dystrophy, and accident-related denervation. The other major finding of the study (i.e. the observation of SSC-induced muscle imbalance in the antagonist TA muscle of mice) confirms the notion that imbalance to phasic muscles can result from exposure of agonist muscle to repeated sessions of contractions. The clinical implication is to utilize exercises which require activation of antagonist muscles for stabilization as a strategy to prevent the onset of muscle imbalance during physical therapy.

## Additional files

Additional file 1: Table S1. Differential expression of genes relevant to myogenesis and muscle growth for PLT and TA muscles following plantarflexion SSC-training relative to non-trained muscles. (DOCX 20 kb)
Additional file 2: Table S2. Differential expression of genes relevant to skeletal muscle wasting and atrophy for PLT and TA muscles following plantarflexion SSC-training relative to non-trained muscles. (DOCX 19 kb)
Additional file 3: Table S3. Differential expression of genes relevant to the titin and dystrophin-glycoprotein complexes for PLT and TA muscles following plantarflexion SSC-training relative to non-trained muscles. (DOCX 19 kb)
Additional file 4: Table S4. Differential expression of genes relevant to energetics and muscle fiber type for PLT and TA muscles following plantarflexion SSC-training relative to non-trained muscles. (DOCX 19 kb)
Additional file 5: Table S5. Top 10 most activated and inhibited biological functions (as predicted by IPA) for PLT and TA muscles following plantarflexion SSC-training relative to non-trained muscles. (DOCX 20 kb)

## Abbreviations

IPA: Ingenuity Pathway Analysis
MHC: Myosin heavy chain
PLT: Plantaris
SSC: Stretch-shortening contraction
TA: Tibialis anterior
