# Supplementary material for: Agonist muscle adaptation accompanied by antagonist muscle atrophy in the hindlimb of mice following stretch-shortening contraction training
Source: BMC Musculoskelet Disord. 2017 Feb 2;18:60. doi: 10.1186/s12891-017-1397-4 (PMC5288976; doi:10.1186/s12891-017-1397-4)
Supplement: Additional file 1: Table S1. — Differential expression of genes relevant to myogenesis and muscle growth for PLT and TA muscles following plantarflexion SSC-training relative to non-trained muscles. (DOCX 20 kb) [file 12891_2017_1397_MOESM1_ESM.docx]

Table S1. Differential expression of genes relevant to myogenesis and muscle growth for PLT and TA muscles following plantarflexion SSC-training relative to non-trained muscles.

|  |  |  |  | PLT | | TA | |
| --- | --- | --- | --- | --- | --- | --- | --- |
|  | Symbol | Description | RefSeq # | Fold change | *P* value | Fold change | *P* value |
| Myogenesis |  |  |  |  |  |  |  |
|  | *Hdac5* | Histone deacetylase 5 | NM_010412 | ↓1.370 | 4.06E-02 | ↓1.545 | 5.87E-02 |
|  | *Myod1* | Myogenic differentiation 1 | NM_010866 | ↓1.469 | 2.08E-02 | ↓1.162 | 9.97E- 01 |
|  | *Pax7* | Paired box gene 7 | NM_011039 | ↑1.427 | 1.68E-02 | ↓1.223 | 4.36E-01 |
|  | *Myf5* | Myogenic factor 5 | NM_008656 | ↑1.345 | 3.20E-02 | ↑1.281 | 2.16E-01 |
|  | *Myog* | Myogenin | NM_031189 | ↑2.679 | 2.34E-03 | ↑9.137 | 1.68E-02 |
|  | *Musk* | Muscle, skeletal, receptor tyrosine kinase | NM_010944 | ↓1.199 | 1.43E-02 | ↑2.655 | 7.28E -03 |
|  | *Lmna* | Lamin A | NM_019390 | ↓1.001 | 7.59E-01 | ↑1.896 | 1.64E-02 |
|  | *Myf6* | Myogenic factor 6 | NM_008657 | ↓1.058 | 5.73E-02 | ↑1.877 | 2.03E-02 |
|  | *Pax3* | Paired box gene 3 | NM_008781 | ↓1.180 | 2.03E-01 | ↓1.803 | 7.71E-03 |
| Muscle autocrine signaling /hypertrophy |  |  |  |  |  |  |  |
|  | *Igf2* | Insulin-like growth factor 2 | NM_010512 | ↑2.558 | 2.32E-03 | ↑1.57 | 1.22E -01 |
|  | *Tgfb1* | Transforming growth factor, beta 1 | NM_011577 | ↑1.369 | 9.51E-03 | ↑2.233 | 1.60E-02 |
|  | *Mstn* | Myostatin | NM_010834 | ↓1.479 | 6.49E-03 | ↓3.106 | 5.64E- 03 |
|  | *Fgf2* | Fibroblast growth factor 2 | NM_008006 | ↓1.803 | 3.06E-03 | ↓3.369 | 4.71E-03 |
|  | *Acvr2b* | Activin receptor IIB | NM_007397 | ↓1.610 | 1.59E-01 | ↓2.934 | 1.07E-02 |

Differential gene expression which surpassed 1.3-fold change with a *P* value < 0.05 were color highlighted; orange – increased expression, blue – decreased expression. Sample sizes were *N* = 8 to 9 per group.
