# Supplementary material for: Agonist muscle adaptation accompanied by antagonist muscle atrophy in the hindlimb of mice following stretch-shortening contraction training
Source: BMC Musculoskelet Disord. 2017 Feb 2;18:60. doi: 10.1186/s12891-017-1397-4 (PMC5288976; doi:10.1186/s12891-017-1397-4)
Supplement: Additional file 2: Table S2. — Differential expression of genes relevant to skeletal muscle wasting and atrophy for PLT and TA muscles following plantarflexion SSC-training relative to non-trained muscles. (DOCX 19 kb) [file 12891_2017_1397_MOESM2_ESM.docx]

|  |  |  |  | PLT | | TA | |
| --- | --- | --- | --- | --- | --- | --- | --- |
|  | Symbol | Description | RefSeq # | Fold change | *P* value | Fold change | *P* value |
| Muscle wasting & atrophy |  |  |  |  |  |  |  |
|  | *Nos2* | Nitric oxide synthase 2, inducible | NM_010927 | ↑1.778 | 4.77E-02 | ↓1.489 | 5.31E-01 |
|  | *Mmp9* | Matrix meallopeptidase 9 | NM_013599 | ↓2.039 | 2.52E-03 | ↓2.724 | 5.12E-02 |
|  | *Akt2* | Thymoma viral proto-oncogene 2 | NM_007434 | ↓1.485 | 4.74E-03 | ↓1.080 | 5.58E- 01 |
|  | *Fbxo32* | F-box protein 32 | NM_026346 | ↓1.349 | 3.28E-02 | ↓1.466 | 1.13E-01 |
|  | *Foxo1* | Foxhead box O1 | NM_019739 | ↓1.370 | 2.06E-02 | ↑1.042 | 5.63E-01 |
|  | *Rps6kb1* | Ribosomal protein S6 kinase, polypeptide 1 | NM_028259 | ↓1.252 | 1.27E-02 | ↓1.545 | 1.27E-02 |
|  | *Ppargc1a* | Peroxisome proliferator activated receptor, gamma, coactivator 1 alpha | NM_008904 | ↓1.161 | 2.53E-01 | ↓1.532 | 8.67E-03 |
|  | *Mapk14* | Mitogen-activated protein kinase 14 | NM_011951 | ↓1.427 | 6.14E-02 | ↓2.045 | 3.68E-02 |
|  | *Casp3* | Caspase 3 | NM_009810 | ↑1.171 | 2.73E-01 | ↑2.627 | 3.73E-02 |

Table S2. Differential expression of genes relevant to skeletal muscle wasting and atrophy for PLT and TA muscles following plantarflexion SSC-training relative to non-trained muscles.

Differential gene expression which surpassed 1.3-fold change with a *P* value < 0.05 were color highlighted; orange – increased expression, blue – decreased expression. Sample sizes were *N* = 8 to 9 per group.
