# Supplementary material for: Agonist muscle adaptation accompanied by antagonist muscle atrophy in the hindlimb of mice following stretch-shortening contraction training
Source: BMC Musculoskelet Disord. 2017 Feb 2;18:60. doi: 10.1186/s12891-017-1397-4 (PMC5288976; doi:10.1186/s12891-017-1397-4)
Supplement: Additional file 3: Table S3. — Differential expression of genes relevant to the titin and dystrophin-glycoprotein complexes for PLT and TA muscles following plantarflexion SSC-training relative to non-trained muscles. (DOCX 19 kb) [file 12891_2017_1397_MOESM3_ESM.docx]

|  |  |  |  | PLT | | TA | |
| --- | --- | --- | --- | --- | --- | --- | --- |
|  | Symbol | Description | RefSeq # | Fold change | *P* value | Fold change | *P* value |
| Titin complex |  |  |  |  |  |  |  |
|  | *Cryab* | Crystallin, alpha B | NM_009964 | ↓1.616 | 2.67E-03 | ↑1.89 | 9.99E-04 |
|  | *Myot* | Myotilin | NM_001033621 | ↓1.330 | 6.92E-03 | ↓1.746 | 7.38E-02 |
|  | *Capn3* | Calpain 3 | NM_007601 | ↓1.542 | 8.68E-03 | ↓2.300 | 1.52E-02 |
|  | *Ttn* | Titin | NM_011652 | ↓1.165 | 6.30E-02 | ↓1.722 | 2.83E-02 |
|  | *Neb* | Nebulin | NM_010889 | ↑1.016 | 6.53E-01 | ↓1.591 | 1.08E-02 |
|  | *Actn3* | Actinin alpha 3 | NM_013456 | ↓1.275 | 2.49E-02 | ↓1.775 | 1.90E-02 |
| Dystrophin-glycoprotein complex |  |  |  |  |  |  |  |
|  | *Camk2g* | Calcium/calmodulin-dependent protein kinase II gamma | NM_178597 | ↓1.299 | 1.92E-02 | ↓1.846 | 1.26E-02 |
|  | *Dag1* | Dystroglycan 1 | NM_010017 | ↓1.240 | 4.02E-02 | ↓1.602 | 2.64E-02 |

Table S3. Differential expression of genes relevant to the titin and dystrophin-glycoprotein complexes for PLT and TA muscles following plantarflexion SSC-training relative to non-trained muscles.

Differential gene expression which surpassed 1.3-fold change with a *P* value < 0.05 were color highlighted; orange – increased expression, blue – decreased expression. Sample sizes were *N* = 8 to 9 per group.
