# Supplementary material for: Agonist muscle adaptation accompanied by antagonist muscle atrophy in the hindlimb of mice following stretch-shortening contraction training
Source: BMC Musculoskelet Disord. 2017 Feb 2;18:60. doi: 10.1186/s12891-017-1397-4 (PMC5288976; doi:10.1186/s12891-017-1397-4)
Supplement: Additional file 4: Table S4. — Differential expression of genes relevant to energetics and muscle fiber type for PLT and TA muscles following plantarflexion SSC-training relative to non-trained muscles. (DOCX 19 kb) [file 12891_2017_1397_MOESM4_ESM.docx]

|  |  |  |  | PLT | | TA | |
| --- | --- | --- | --- | --- | --- | --- | --- |
|  | Symbol | Description | RefSeq # | Fold change | *P* value | Fold change | *P* value |
| Energy metabolism / diabetes & metabolic syndrome |  |  |  |  |  |  |  |
|  | *Hk2* | Hexokinase 2 | NM_013820 | ↓1.571 | 1.98E-02 | ↓1.647 | 5.25E-02 |
|  | *Pdk4* | Pyruvate dehydrogenase kinase, isoenzyme 4 | NM_013743 | ↓1.307 | 4.73E-02 | ↓1.383 | 4.58E-02 |
|  | *Prkab2* | Protein kinase, AMP-activated, beta 2 non catalytic subunit | NM_182997 | ↓1.507 | 4.07E-03 | ↓3.234 | 6.96E- 04 |
|  | *Slc2a4* | Solute carrier family 2 (facilitated glucose transporter), member 4 | NM_009204 | ↓1.348 | 1.40E-02 | ↓2.275 | 1.42E-02 |
| Skeletal muscle fiber type |  |  |  |  |  |  |  |
|  | *Myh1* | Myosin, heavy polpeptide 1, skeletal muscle, adult | NM_030679 | ↑1.589 | 2.66E-03 | ↑1.028 | 7.93E-01 |
|  | *Tnni2* | Tropinin I, skeletal, fast 2 | NM_009405 | ↓1.432 | 4.73E-06 | ↓1.846 | 1.26E- 02 |
|  | *Tnnt3* | Troponin T3, skeletal, fast | NM_011620 | ↓1.368 | 5.45E-04 | ↓1.700 | 2.19E-03 |
|  | *Myh2* | Myosin, heavy polypeptide 2, skeletal muscle, adult | NM_001039545 | ↓1.253 | 1.87E-02 | ↓2.605 | 2.00E-02 |
|  | *Mb* | Myoglobin | NM_013593 | ↓1.287 | 2.84E-02 | ↑1.708 | 1.54E-02 |

Table S4. Differential expression of genes relevant to energetics and muscle fiber type for PLT and TA muscles following plantarflexion SSC-training relative to non-trained muscles.

Differential gene expression which surpassed 1.3-fold change with a *P* value < 0.05 were color highlighted; orange – increased expression, blue – decreased expression. Sample sizes were *N* = 8 to 9 per group.
