# Supplementary material for: Agonist muscle adaptation accompanied by antagonist muscle atrophy in the hindlimb of mice following stretch-shortening contraction training
Source: BMC Musculoskelet Disord. 2017 Feb 2;18:60. doi: 10.1186/s12891-017-1397-4 (PMC5288976; doi:10.1186/s12891-017-1397-4)
Supplement: Additional file 5: Table S5. — Top 10 most activated and inhibited biological functions (as predicted by IPA) for PLT and TA muscles following plantarflexion SSC-training relative to non-trained muscles. (DOCX 20 kb) [file 12891_2017_1397_MOESM5_ESM.docx]

|  | Function | Activation z-score | *P* value |
| --- | --- | --- | --- |
| PLT |  |  |  |
|  | Hypertrophy | 2.464 | 2.18E-11 |
|  | Fusion of cells | 2.228 | 2.14E-10 |
|  | Concentration of hormone | 2.200 | 1.63E-05 |
|  | Necrosis of epithelial tissue | 2.126 | 1.84E-05 |
|  | Hypertrophy of heart | 2.009 | 4.83E-10 |
|  | Cardiogenesis | 2.000 | 2.73E-08 |
|  | Quantity of insulin in blood | 1.968 | 1.19E-04 |
|  | Quantity of muscle cells | 1.965 | 2.67E-07 |
|  | Fibrosis of heart | 1.910 | 8.44E-05 |
|  | Concentration of eicosanoid | 1.859 | 1.12E-05 |
|  | Tubulation of endothelial cells | -1.186 | 1.23E-05 |
|  | Differentiation of connective tissue cells | -1.246 | 9.33E-10 |
|  | Differentiation of fibroblast cell lines | -1.412 | 2.44E-06 |
|  | Size of bone | -1.457 | 8.71E-08 |
|  | Glycolysis | -1.498 | 6.43E-07 |
|  | Blood pressure | -1.513 | 5.89E-05 |
|  | Catabolism of protein | -1.710 | 1.41E-06 |
|  | Fibrosis of muscle | -1.715 | 6.91E-08 |
|  | Quantity of connective tissue | -1.756 | 1.26E-07 |
|  | Quantity of adipose tissue | -1.945 | 3.49E-05 |
| TA |  |  |  |
|  | Quantity of muscle cells | 1.896 | 5.00E-07 |
|  | Cell death | 1.703 | 2.83E-07 |
|  | Glucose metabolism disorder | 1.692 | 3.32E-04 |
|  | Atrophy of muscle | 1.654 | 1.78E-09 |
|  | Differentiation of muscle cell lines | 1.513 | 1.11E-07 |
|  | Hyperglycemia | 1.477 | 6.08E-06 |
|  | Cell death of neuroglia | 1.452 | 5.85E-07 |
|  | Mass of heart | 1.387 | 1.12E-06 |
|  | Apoptosis of neurons | 1.373 | 6.38E-05 |
|  | Differentiation of muscle | 1.359 | 1.24E-14 |
|  | Differentiation of fibroblast cell lines | -1.412 | 4.54E-06 |
|  | Proliferation of embryonic cells | -1.412 | 5.21E-04 |
|  | Mass of hindlimb muscle | -1.539 | 1.78E-07 |
|  | Mass of skeletal muscle | -1.713 | 1.43E-11 |
|  | Angiogenesis | -1.786 | 1.89E-05 |
|  | Cell cycle progression | -1.794 | 7.22E-06 |
|  | Vasculogenesis | -1.802 | 2.98E-05 |
|  | Function of skeletal muscle | -1.840 | 4.84E-16 |
|  | Proliferation of cells | -1.854 | 2.05E-04 |
|  | Transport of D-glucose | -1.931 | 1.71E-06 |

Table S5. Top 10 most activated and inhibited biological functions (as predicted by IPA) for PLT and TA muscles following plantarflexion SSC-training relative to non-trained muscles.

All IPA functions with the exception of cancer specific functions were considered for this listing.
